# COmmunity-based Rehabilitation after Knee Arthroplasty (CORKA): study protocol for a randomised controlled trial

**DOI:** 10.1186/s13063-016-1629-1

**Published:** 2016-10-13

**Authors:** Karen L. Barker, David Beard, Andrew Price, Francine Toye, Martin Underwood, Avril Drummond, Gary Collins, Susan Dutton, Helen Campbell, Nicola Kenealy, Jon Room, Sarah E. Lamb

**Affiliations:** 1 NIHR – BRU, Nuffield Department of Orthopaedics, Rheumatology and Musculoskeletal Sciences, University of Oxford, Oxford, OX3 7LD UK; 2 Physiotherapy Research Unit, Nuffield Orthopaedic Centre, Oxford University Hospitals NHS Foundation Trust, Windmill Road, Oxford, OX3 7HE UK; 3 Division of Health Sciences, Warwick Medical School, University of Warwick, Coventry, CV4 7AL UK; 4 Faculty of Medicine and Health Sciences, University of Nottingham, Nottingham, NG7 2UH UK; 5 Centre for Statistics in Medicine, University of Oxford, Oxford, OX3 7LD UK; 6 Health Economics Research Centre, Department of Public Health, University of Oxford, Oxford, OX3 7LF UK

**Keywords:** Randomised controlled trial, Knee arthroplasty, Physiotherapy, Occupational therapy, Rehabilitation, Community, Elderly, Frail

## Abstract

**Background:**

The number of knee arthroplasties performed each year is steadily increasing. Although the outcome is generally favourable, up to 15 % fail to achieve a satisfactory clinical outcome which may indicate that the existing model of rehabilitation after surgery may not be the most efficacious. Given the increasing number of knee arthroplasties, the relative limited physiotherapy resources available and the increasing age and frailty of patients receiving arthroplasty surgery, it is important that we concentrate our rehabilitation resources on those patients who most need help to achieve a good outcome. This pragmatic randomised controlled trial will investigate the clinical and cost-effectiveness of a community-based multidisciplinary rehabilitation intervention in comparison to usual care.

**Methods/design:**

The trial is designed as a prospective, single-blind, two-arm randomised controlled trial (RCT). A bespoke algorithm to predict which patients are at risk of poor outcome will be developed to screen patients for inclusion into a RCT using existing datasets. Six hundred and twenty patients undergoing knee arthroplasty, and assessed as being at risk of poor outcome using this algorithm, will be recruited and randomly allocated to one of two rehabilitation strategies: usual care or an individually tailored community-based rehabilitation package. The primary outcome is the Late Life Function and Disability Instrument measured at 1 year after surgery. Secondary outcomes include the Oxford Knee Score, the Knee injury and Osteoarthritis Outcome Score quality of life subscale, the Physical Activity Scale for the Elderly, the EQ-5D-5L and physical function measured by three performance-based tests: figure of eight, sit to stand and single-leg stand. A nested qualitative study will explore patient experience and perceptions and a health economic analysis will assess whether a home-based multidisciplinary individually tailored rehabilitation package represents good value for money when compared to usual care.

**Discussion:**

There is lack of consensus about what constitutes the optimum package of rehabilitation after knee arthroplasty surgery. There is also a need to tailor rehabilitation to the needs of those predicted to do least well by focussing on interventions that target the elderly and frailer population receiving arthroplasty surgery.

**Trial registration:**

ISRCTN 13517704, registered on 12 February 2015.

## Background

The number of knee arthroplasty (KA) operations taking place in the UK is continuing to rise; 96,986 primary KAs were recorded in 2014, a 12.9 % increase over 2013 with osteoarthritis the most common indication for surgery [1]. Large numbers of KAs are being performed in older and frailer patients. In 2014, 18 % of operations were performed on patients with a patient physical status recorded as ‘incapacitating systemic disease’ (P3 or greater), with the 99^th^ percentile age being between 85 and 88 years and the oldest patient being 101 years [1]. The existing literature demonstrates that predicting who will do well after KA is a complex construct and not determined by simplistic linear relationships with factors such as age or presurgical function. A number of studies have investigated the influence of preoperative predictors on postoperative outcome of KA. However, no screening algorithm that can accurately identify and predict who is at a risk of poor postoperative outcome associated with rehabilitation is currently in existence. Generally, patients who are better preoperatively tend to have a better postoperative outcome [2–5]. Evidence on the influence of comorbidities on postoperative outcome is inconclusive. A number of studies demonstrate the association of preoperative comorbidities with a worse postoperative outcome [4, 6, 7] but others do not observe such an association [3, 8]. Age, however, should not be a barrier to having a good outcome from KA, with reports of a successful outcome in patients aged over 80 years [9].

Furthermore, it is known that outcome following KA is multifaceted; around 15 % of patients do not report a good outcome following their KA and have continuing pain and mobility problems which limit or prevent them from doing activities they would like to do after surgery [10]. Factors such as the amount of pain and limitation of balance and muscle strength may contribute to poorer outcome [11] and effective rehabilitation interventions may contribute to optimising postoperative return to functional activities [12].

### Rehabilitation approaches

Systematic reviews evaluating the effectiveness of exercise support the use of functional physiotherapy exercise interventions following discharge to obtain short-term benefit following elective primary KA [12, 13]. These reviews revealed the complexity involved in deciding the best rehabilitation after KA. The lack of knowledge regarding current physiotherapy practice has been recognised internationally [14], with no generally accepted rehabilitation protocol for patients post KA. A recent review examined multidisciplinary rehabilitation programmes following hip and knee joint arthroplasty and, although it concluded that home-based care may be beneficial, stressed the low quality of the current evidence base and concluded that further high-quality research is needed [15]. Moreover, there are no published randomised controlled trials (RCTs) of occupational therapy after KA and many published studies either have serious methodological limitations or it is difficult to extrapolate the contribution of occupational therapy from the overall rehabilitation package.

In the UK, Clinical Commissioning Groups typically will fund four to six sessions of outpatient postoperative physiotherapy [16], however, previous research has shown that this short course of physiotherapy is not needed by all patients to help them recover after surgery [17]. Conversely, concern has been raised that many exercise programmes lack adequate intensity to lead to optimal recovery [18, 19]. Internationally, where much greater doses of physiotherapy are often provided, research indicates that 12–18 h of physiotherapy [20], or a mean of 17 visits [21], may be needed to produce benefit. These levels of care may be well beyond those provided in the UK and, in the current economic climate, may be more than the NHS can afford given the numbers of KAs undertaken each year. Given the increasing number of KAs, the relatively limited physiotherapy resources available and the increasing age and frailty of patients receiving joint arthroplasty, it is important that we concentrate our rehabilitation resources on those patients who need most help to avoid a poor outcome.

Current evidence suggests an optimal rehabilitation approach should include a structured programme that incorporates muscle-strengthening exercises, including resistive muscle-strengthening exercises which are regularly progressed along with exercises to improve balance. Exercises which facilitate an improvement or maintenance of daily living activities, such as housework and personal care activities, plus endurance exercises to improve baseline levels of physical activity are also required as overall health permits [22–25]. It is also imperative that exercise and functional rehabilitation are linked to demonstrable increases in activity output and participation levels.

Many patients may also benefit from environmental modifications, aids and appliances where impairments cannot be overcome or as part of the therapeutic programme to increase their functional performance; these will be provided where needed as part of the intervention. Our approach is to include exercises and activities which address more than one aim, are progressed in difficulty and are individually tailored to each patient to maximise their performance. The intervention in this study also needs to be manageable for older patients, who may be frail and with significant comorbidities and for whom the 60–90-min intensive sessions recommended following total KA [23] are unachievable.

The programme must also be attentive to the need to develop an intervention that can translate into routine clinical practice and be affordable to health care commissioners.

In view of this we will develop an intervention that is staffed by both qualified physiotherapists and rehabilitation assistants. Rehabilitation assistants are routinely used in the delivery of exercise programmes to patients following orthopaedic surgery and we will test the safety and efficacy of this model of delivery.

We have selected to test a combined physiotherapy- and occupational therapy-informed intervention delivered in patients’ homes. The full details of the intervention will be published separately in accordance with recent TIDieR guidance [26].

### Objectives

1. To design a prognostic screening algorithm which will be developed based on an analysis of factors associated with poor outcome following KA
2. To evaluate, in a population identified as at risk of poor outcome, if a multicomponent rehabilitation programme delivered in patients’ homes can improve their outcome compared to those receiving the standard outpatient rehabilitation over a 12-month period
3. To undertake a nested qualitative study exploring the perceptions of both patients and clinicians on the use of the community-based rehabilitation programme
4. To undertake an economic analysis to compare the cost-effectiveness of both the intervention and usual care

## Methods/design

### Trial design

COmmunity-based Rehabilitation after Knee Arthroplasty (CORKA) is a prospective, individually randomised controlled trial with blinded outcome assessment for the clinical outcomes at baseline, 6 and 12 months. It will also include a nested qualitative study and a health economic analysis. The trial was preceded by a development phase where we designed a screening tool by analysing data from existing NHS datasets from the KAT trial [27] to develop an algorithm to be used at preoperative assessment to identify patients likely to be at risk of poor outcome after KA. The screening tool was developed and internally validated prior to the recruitment of the first patient into the trial. A manuscript describing the development and internal validation of the screening tool is currently being prepared for submission.

**Fig. 1:**
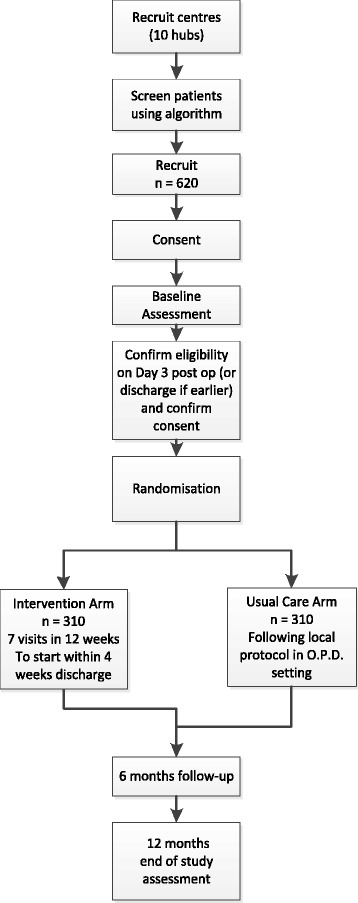
Study flow chart

Patients will be randomised to either usual care (control) or to a community-based intervention group. Baseline assessments will be collected no more than 4 weeks before participants’ date of surgery. Follow-up assessment will take place at 6 and 12 months after randomisation. The protocol conforms to the Standard Protocol Items: Recommendations for Interventional Trials (SPIRIT) guidelines for nonpharmacological studies [28] (Fig. 1).

### Study population

Six hundred and twenty patients at risk of poor outcome, identified through the screening algorithm will be recruited to the study. The planned recruitment period is 18 months.

### Eligibility criteria

#### Inclusion criteria

- Participant is willing and able to give informed consent for participation in the study
- Men or women, aged 55 years or above
- Primary unilateral KA as a scheduled procedure
- At risk of poor outcome – as identified by the study screening tool
- Willing to allow rehabilitation teams to attend their home to deliver the community-based rehabilitation programme if randomised to the intervention arm

#### Exclusion criteria

- Any absolute contraindications to exercise
- Severe cardiovascular or pulmonary disease (New York Heart Association classes III–IV)
- Severe dementia, assessed using the hospital dementia screening tool
- Rheumatoid arthritis (active disease)
- Further lower limb arthroplasty surgery planned within 12 months.
- Serious perioperative complications

### Procedures

#### Recruitment

A minimum of six and up to ten NHS hospitals that carry out elective primary KA will participate to recruit 620 participants. People who are scheduled to receive a knee replacement will be invited to take part in the trial once they have been be assessed for likelihood of poor outcome using the screening tool developed as part of this study. This will be administered in the preoperative assessment clinic and the data will be screened for study suitability by a member of the local team. Baseline assessments will be collected no longer than 4 weeks before participants’ date of surgery. Follow-up assessment will take place at 6 and 12 months after randomisation.

### Randomisation, blinding and allocation concealment

The final decision about inclusion and recruitment to the trial will be made at day 3 post operatively, when patients may be excluded if they have had any serious perioperative complications; or on discharge from hospital if before day 3.

If eligible, participants will have their consent confirmed and randomisation will take place using a website-based system provided by the Oxford Clinical Trials Research Unit randomisation service. Randomisation uses permuted blocks of random and undisclosed sizes stratified by site. Participants will be allocated to receive one of two rehabilitation options, either ‘usual care’ or the ‘home-based exercise programme’. The research therapist at each site will then be informed of the participant’s treatment allocation and will liaise with the appropriate clinical staff to provide the correct intervention.

Due to the nature of the intervention participants and those delivering the rehabilitation will be aware of the treatment allocation; by virtue of the design it is not possible to blind the participants or physiotherapists [29].

Follow-up assessments will be performed by a blinded research physiotherapist and the staff recruiting participants and performing baseline and follow-up assessments will not be involved in delivering the treatment interventions. All data will be entered by a data entry assistant to ensure that the research physiotherapists remain blind to treatment allocation. All outcome assessors will remain blinded until the final analysis is complete. We will use the methods described by Minns Lowe et al. to assess the success of assessor blinding [29].

### Outcome measures

#### Primary outcome measure

The Late Life Function and Disability Instrument (LLFDI) is the primary outcome assessment. It is a 48-item outcome instrument developed and validated specifically for community-dwelling older adults, which assesses and responds to meaningful change in two distinct domains: function; a person’s ability to do discrete actions or activities, and disability; and a person’s performance of socially defined life tasks [30, 31]. The LLFDI will be reported by the aggregated function and disability component scores as well as a whole to correspond to the International classification of functioning, disability and health (ICF). The total score will be the primary outcome.

#### Secondary outcome measures

The Oxford Knee Score (OKS). This is a disease-specific measure to assess function and to allow comparison with data from large epidemiological cohort studies. It is a 12-item patient-reported outcome measure which measures pain and function after KA surgery [32].

The Knee injury and Osteoarthritis Outcome Score (KOOS) quality of life subscale. The KOOS is a specifically validated instrument developed for knee osteoarthritis, which can also be analysed to calculate a Western Ontario and McMaster Universities (WOMAC) Index. It is a self-reported questionnaire consisting of five subscales: pain, other symptoms, activities of daily living (ADL), function in sport and recreation (sport/rec) and knee-related quality of life (QOL). The quality of life subscale of the KOOS, consists of four self-reported questions [33].

The Physical Activity Scale for the Elderly (PASE) questionnaire. A self-reported scale designed to measure the physical activity level of those aged 65 years and older. It consists of three subscales, leisure time activity, household activity and work-related activity. This is a short, self-administered questionnaire to assess activity in the past week [34].

Health economics using the EuroQol 5 dimensions, 5 levels questionnaire (EQ-5D-5L). A validated self-reported outcome measure consisting of five dimensions: mobility, self-care, usual activity, pain and discomfort and anxiety and depression. Each dimension has five categories of response. It is designed to provide a generic measure of health status for clinical and/or economic evaluation [35].

Functional Co-morbidities Index. This will be completed as other diseases are likely to be present in this older population which might affect physical outcomes [36].

Physical measures. Measures of outcome include measures of balance, mobility and physical activity, all areas affected by KA. Each test is reliable and valid, has been used with older, community-dwelling adults and has been shown to be responsive in previous rehabilitation studies. Physical function will be measured by three physical performance tasks: the Figure of 8 walk test, the 30-s chair-stand test and the single-leg stance. [37–39].

All data will be collected by face-to-face clinical assessment at baseline and 6 and 12 months post randomisation [Table 1].

**Table 1:** Summary of outcomes and assessment schedule

Time point	Enrolment/Pre op clinic	Baseline	Surgery	Allocation	Post surgery			
					Weeks 0–2	Weeks 3–6	6 months	12 months
KA surgery			X					
High-risk screening tool applied	X							
Eligibility screen: inclusion/exclusion criteria	X							
Informed consent	X							
Baseline questionnaire		X						
Randomisation				X				
Intervention arm: home-based exercise programme					X			
Usual care arm: routine care, local centre					X			
Assessments								
• Demographics • Medical History • EQ-5D-5L presurgery recall		X X X						
• LLFDI score • Oxford Knee Score • Quality of life subscale of the Knee Osteoarthritis Outcome Score (KOOS) • Physical Activity Scale for the Elderly (PASE) questionnaire. • Health economics using the EQ-5D-5L • 30-s chair-stand test • Figure of 8 walk test • Single-leg stance		X X X X X X X					X X X X X X X	X X X X X X X
Participant Diary: completed daily/as required at home for 6 weeks Then weekly recording: • Exercises undertaken • Medication taken • Use of health care services and personnel					X	X	X	X
Adverse events Collected throughout			X		X	X	X	X

This table excludes the qualitative substudy taking place in selected sites/participants

*EQ-5D-5L* EuroQol 5 dimensions, 5 levels questionnaire, *KA* knee arthroplasty, *LLFDI* Late Life Function and Disability Instrument

### Interventions

#### Usual care arm

Those in the usual care arm will receive the routine care offered by the local centre. This is likely to include written advice on home exercises provided on discharge from hospital; between 1 and 6 sessions of traditional outpatient physiotherapy and home requirements assessed by an occupational therapist to identify barriers to discharge. It is recognised that usual care can vary geographically [16] and this may include the number of sessions of physiotherapy given post discharge. To standardise the usual care arm, as far as possible there will be a minimum and maximum number of session that will be included in usual care. Participants will be expected to attend at least one session of outpatient physiotherapy and no more than six sessions.

#### Intervention arm

The intervention is a multicomponent rehabilitation programme designed to improve both the function of ‘at risk’ patients and their participation in activities. The largest component will be an exercise programme, delivered in the participants’ own homes, in order to make it accessible to those without good social support or those with physical or mental frailty. The programme will consist of an individualised set of exercises covering exercises selected from a menu to include at least one exercise from each of the following sections: knee flexion, knee extension, knee-strengthening, hip-strengthening, static balance and gait skills. Attention will also be paid to pain management, confidence-building, appropriate provision of aids and equipment and suitability of the home environment. In order to make the intervention affordable to the NHS, the trial will use a combination of qualified physiotherapists, occupational therapists and rehabilitation assistants to deliver the intervention. The programme will focus on both improving functional outcome but also on participation levels.

Collaborating sites will provide the CORKA home-based rehabilitation programme. It will commence delivery within 4 weeks of KA surgery. (A window of 2–8 weeks for starting the intervention will be allowed before a protocol deviation is considered to have occurred.)

#### Governance

The sponsor of the trial is the University of Oxford and the University’s Clinical Trials and Research Office (CTRG) will oversee the roles and responsibilities delegated to them as research sponsor.

Trial Steering Committee (TSC): the TSC, which includes independent members, provides overall supervision of the trial on behalf of the funder. The terms of reference are agreed with the Health Technology Assessment (HTA) and drawn up in a TSC charter which outlines its roles and responsibilities. Meetings of the TSC will take place at least once a year during the recruitment period.

Trial Management Group (TMG): the TMG is made up of the investigators listed on the front of this protocol, and staff working on the project within the Oxford Clinical Trials Research Unit (OCTRU)/CCTR Trials Group. This group will oversee the day-to-day running of the trial and will meet regularly throughout the lifetime of the study.

Data Monitoring and Safety Committee (DMSC): the DMSC is a group of independent experts external to the trial who assess the progress, conduct, participant safety and, if required, critical endpoints of a clinical trial. The DMSC will adopt a DAMOCLES charter which defines its terms of reference and operation in relation to trial oversight [40]. It will not be asked to perform any formal interim analyses of effectiveness. It will, however, see copies of data accrued to date, or summaries of that data by treatment group and will assess the screening algorithm against the eligibility criteria. It will also consider emerging evidence from other related trials or research and review-related serious adverse events (SAEs) that have been reported. It may also advise the chair of the TSC at any time if, in its view, the trial should be stopped for ethical reasons, including concerns about participant safety. DMSC meetings will be held at least annually during the recruitment phase of the study.

All data and documentation will be stored in accordance with regulatory requirements regarding confidentiality and access to the data will be restricted to authorised trial personnel. The Oxford Clinical Trials Research Unit will securely hold the database.

### Reporting of adverse events

Full definitions of SAEs, foreseeable adverse events and the mechanisms for reporting and assessing adverse events are given in the full protocol available on line. A SAE is any untoward medical occurrence that: (1) results in death, (2) is life-threatening, (3) requires inpatient hospitalisation or prolongation of existing hospitalisation, (4) results in persistent or significant disability/incapacity or (5) consists of a congenital anomaly or birth defect. Other ‘important medical events’ may also be considered serious if they jeopardise the participant or require an intervention to prevent one of the above consequences.

#### Foreseeable adverse events

Fall risk is an important issue as this population is at higher risk for falls; whether the home exercise group is at higher risk is debatable but needs consideration and so will be carefully monitored. The following data will be collected and recorded in the Participant Diary:

- A fall in the home: during active delivery of the home-based rehabilitation programme that does not meet the criteria of a SAE as above
- A fall in the home: at any time outside of the delivery of the home-based rehabilitation programme
- A fall in the garden at home
- A fall at any other location/outside of the home environment

Falls which are assessed as being related to the study intervention and are categorised as serious according to the listed definitions for a serious adverse event, will also be recorded and reported to the trial office using an SAE Form.

Other foreseeable adverse events: some adverse events will be expected as part of the surgery received rather than inclusion in the CORKA study/receiving rehabilitation. These will be collected as part of standard data collection on the study questionnaires/Case Report Forms (CRFs) but are not classified as reportable SAEs:

- Infection of knee replacement
- Fracture
- Venous thromboembolism/pulmonary embolism

The trials office will be responsible for reporting all study SAEs occurring to a participant to the Research Ethics Committee (REC), which gave a favourable opinion of the study, where the event is confirmed as ‘serious’, ‘related’ and ‘unexpected’. The information provided to the REC will be unblinded and will be reported within 15 days of the trial office being made aware.

### Quality monitoring

The trial will be conducted according to the Standard Operating Procedures (SOPs) of the OCTRU. There will be standardised initial training to all CORKA trial assessors and clinical staff involved in delivering the interventions at all sites. After the training the following procedures will be used to promote consistency and high-quality trial procedures across all sites:

- A member of the CORKA team will observe each assessor perform at least one of their assessments to ensure that they take place as per protocol. Repeat visits will be undertaken should any concerns arise until reliable and valid assessments occur
- Within 2 weeks copies of all assessment forms will be sent to the trial office for review in order to identify any issues concerning missing data or poorly completed forms. Any issues or concerns will be discussed with individual assessors
- A member of the CORKA team will check each site’s trial master file and meet with researchers and clinicians from each site on an annual basis (or more frequently should this be necessary)

### Intervention compliance

Compliance with the test intervention will be defined as fulfilling at least four treatment sessions. The number of physiotherapy visits and the content of the treatment sessions will be recorded using clinician-completed treatment logs and patient exercise and participation diaries. A member of the CORKA team will observe clinical staff perform one of their treatments to ensure that all treatments adhere to the protocol. Clinical staff will be asked to complete a treatment log for each attendance, providing an approximate estimation of the time spent on key intervention components and detailing and explaining any deviations from the protocol. Clinician compliance to the treatment protocol will be assessed and monitored by analysis of the treatment logs and site monitoring visits.

### Retention of participants

The study has two follow-up time points, at 6 and 12 months post randomisation. Follow-up can take place at the participant’s own home or at the hospital, depending on where the baseline took place, i.e. the location is consistent at participant level. Local site staff will organise the follow-up and liaise directly with the participant to organise the follow-up visits. Once confirmed, an appointment reminder letter or email can be sent out to the participants with the appropriate questionnaire.

An intention-to-treat analysis will be carried out; therefore, all participants remain in the study irrespective of whether they receive or continue with their allocated treatment (unless the participants themselves withdraw consent).

### Health economics

Health economics analysis will compare the cost-effectiveness over 1 year of providing the community-based intervention against standard care. The economic evaluation will take the form of a cost-utility analysis from a societal perspective and quality-adjusted life years (QALYs) will be used as the main health outcome measure. A micro-costing approach will be used to calculate costs of the home-based rehabilitation intervention and data will be collected from each trial participant on NHS and social care contacts up to 12 months through the use of a Participant Diary. Assessments of the health-related quality of life (HRQoL) of participants in each arm of the trial will be conducted using the EQ-5D-5L instrument at baseline, 6 and 12 months. Utility values derived from the EQ-5D-5L data will be combined with patient survival data and used to estimate QALYs for each patient up to 12 months.

Mean (standard deviation) costs and QALYs per patient will be estimated for each arm of the trial. The difference (95 % confidence interval (CI)) in mean costs and QALYs between trial arms will be estimated and, if necessary, an incremental cost-effectiveness ratio (ICER) calculated to determine the additional cost of generating one additional QALY. Results will be presented from an NHS, patient and societal (including informal care) perspective as recommended by current guidance. Cost-effectiveness acceptability curves will be used to determine the probability that the home rehabilitation programme is cost-effective at different values of society’s willingness to pay per QALY.

### Qualitative study

The nested qualitative study will provide a picture of the issues facing people with an expected poorer outcome after KA – particularly around expectations of outcome rehabilitation and outcomes on the level of function, activity and participation.

The qualitative study will take place in selected sites for a small number of participants (approximately 15 participants, in addition to approximately 15 members of staff). This number of participants is consistent with qualitative methodology. There will be a separate consent process before the interviews are carried out. Recruitment will take place throughout the study to ensure a spread of participants that is representative of the recruited population. Purposive sampling will be used to achieve a sample of participants which includes: female and male participants, those with differing levels of function and disability selected using their baseline LLFDI score and patients of varying activity levels. In addition, a sample of clinical staff who have delivered the intervention from differing professional backgrounds, physiotherapists, occupational therapists and rehabilitation assistants, will be interviewed.

Participants will be invited to take part in in-depth semistructured interviews following the intervention. Interviews will be held at a convenient time and location for each participant, from previous experience this is most likely to be at participant’s homes. The development of the interview schedule will be iterative and the questions asked may develop and change as the interviews are conducted. The interviews will be digitally recorded and fully transcribed. Field notes and memos will be recorded using a digital notepad. Audio recordings will be transcribed verbatim and coded. Interview data will be analysed using Smith’s experiential approach of Interpretative Phenomenological Analysis [41]. NVivo software will be used to assist in managing and presenting the findings. Participants will be offered the opportunity to view a summary of their results, providing an opportunity for them to contribute any additional comments. The research team will discuss the development of themes as the research progresses with the aim of providing different perspectives and enhancing the development of the themes.

### Data and statistical analysis

#### Sample size

Since the LLFDI has not been used widely there is currently little information from the existing literature about the value for the minimal clinically important difference or about the likely treatment difference in LLFDI component scores for this type of study. Therefore, our sample size calculation is based on a moderately small standardised effect size of 0.275, which is a value that we expect to be clinically important and associated with small but worthwhile benefits in rehabilitation trials. Six hundred and twenty participants (310 per arm) are required to detect a standardised effect size of 0.275 with 90 % power, 5 % (two-sided) significance and allowing 10 % loss to follow-up. This standardised effect size is equivalent to detecting around a 3-point difference on the LLFDI between treatment arms assuming a standard deviation of 10.91. The DMSC will be reviewing this assumption and monitoring the standard deviation.

Fifteen participants were randomised during an internal pilot study and will be used in the final analysis.

### Statistical analysis

The study will be reported according to the Consolidated Standards of Reporting Trials (CONSORT) 2010 Statement utilising the nonpharmacological treatment interventions and patient-reported outcome extensions [42, 43].

The principal comparisons will be performed on an intention-to-treat basis. The results from the trial will be presented as comparative summary statistics (i.e. difference in means) with 95 % CIs. The primary outcome will be analysed using a linear mixed effects method with repeated measures, on outcome measurements at 6 and 12 months, adjusting for baseline score and stratification/variables. An interaction between time and randomised group will be fitted to allow estimation of treatment effect at each time point. We will formally assess the distribution of the change from baseline for evidence of departure from normality. If necessary, data will either be transformed or analysed using a nonparametric equivalent. Similar approaches will be carried out for other continuous outcomes.

The nature and mechanism for the missing outcomes will be investigated, though mixed effects models that implicitly account for data following a missing-at-random mechanism. Sensitivity analyses will be carried out to examine the robustness of the results with different assumptions being made about departures from randomisation policies and handling of missing data.

A Statistical Analysis Plan (SAP) containing a more detailed account of the proposed statistical analysis will be drafted early in the trial and approved by the Independent Monitoring Committee for the trial prior to the primary analysis data lock and prior to the randomisation data being added to the database. The data analysis plan will consider in detail the need for baseline covariate adjustment. Any changes at this time will be incorporated into the final SAP and signed off as per current OCTRU Standard Operating Procedures (SOPs). Any changes/deviations from the original SAP will be described and justified in the protocol and/or in the final report, as appropriate.

The trial will be deemed a success based on the primary outcome of the total LLFDI based on the *p* value and if the lower bound of the 95 % CI is greater than 3 points.

Complier-average causal effect (CACE) will be estimated to assess the impact of the intervention compliance on the effect of the interventions.

### Timeline

The trial is funded to run over a period of 51 months and commenced in August 2014. Data analysis, economic analysis and report writing is expected to take place from month 45 onwards (May 2018).

### Dissemination

The chief investigator will coordinate dissemination of data from this study. All publications using data from this study to undertake original analyses will be submitted to the DSMC, TMG and TSC for review before release. The final study report will be available on the HTA website.

We will provide all participants with a summary of the trial outcome.

## Discussion

We have chosen to focus our intervention as a community home-based treatment package. We believe this to be particularly suited to those patients likely to find it hard to access traditional physiotherapy because of transport difficulties, social isolation, frailty and low self-efficacy. For many people relearning daily living skills within their own home and immediate home environment is both desirable and essential.

Given the increasing number of KAs, the relative limited therapy resource available and the increasing age and frailty of patients receiving joint arthroplasties, it is important that we concentrate our rehabilitation resources on those patients who need most help to achieve a good outcome. Furthermore, it is clear from the existing studies that current rehabilitation strategies do not meet the needs of all patients, particularly those who are older and frailer. Addressing the needs of these patients is particularly crucial because all patients are being discharged home earlier from the acute setting, meaning that less time is available for acute physical recovery, rehabilitation and education; thus, the potential burden of care for these patients and their families is increased. This is a particular concern given both the projected increased need for joint arthroplasty over the next decade to accommodate an ageing population and the pressure of potential reductions in NHS funding.

### Trial status

The first patient was randomised to the trial in March 2015. Recruitment for the study is ongoing and currently stands at 210 at the end of August 2016.

This paper is based upon the latest version of the protocol v3 November 2015. In addition to this paper, updated versions of the protocol if amended throughout the trial will be available on the trial website http://corka.octru.ox.ac.uk/welcome-corka-trial and will follow SPIRIT 2013 guidelines [27].

## Abbreviations

AE: Adverse Event
CCTR: Critical Care, Trauma and Rehabilitation Trials Group (part of OCTRU)
CI: Chief Investigator
CRF: Case Report Form
CTRG: Clinical Trials & Research Governance, University of Oxford
GCP: Good Clinical Practice
GP: General Practitioner
ICF: Informed Consent Form
ICH: International Conference of Harmonisation
ISF: Investigator Site File
KR: Knee Replacement
NHS: National Health Service
NRES: National Research Ethics Service
OCTRU: Oxford Clinical Trials Research Unit
PI: Principal Investigator
PIL: Patient Information Leaflet
R&D: NHS Trust R&D Department
RCT: Randomised Controlled Trial
REC: Research Ethics Committee
SAE: Serious Adverse Event
SOP: Standard Operating Procedure
KAT: Knee Arthroplasty Trial
QALY: Quality Adjusted Life Years
